# Dog survey in Russian veterinary hospitals: tick identification and molecular detection of tick-borne pathogens

**DOI:** 10.1186/s13071-018-3161-5

**Published:** 2018-11-14

**Authors:** Natalia N. Livanova, Natalia V. Fomenko, Ivan A. Akimov, Mikhail J. Ivanov, Nina V. Tikunova, Rob Armstrong, Sergey V. Konyaev

**Affiliations:** 1 Institute of Systematics and Ecology of Animals, Siberian Branch of the Russian Academy of Sciences, Frunze, 11, 630091 Novosibirsk, Russia; 2 AO Vector-Best, P.O. Box 492, 630117 Novosibirsk, Russia; 3 Institute of Molecular and Cellular Biology of the Siberian Branch of the Russian Academy of Sciences, Acad. Lavrentiev Ave. 8/2, 630090 Novosibirsk, Russia; 4 MSD Animal Health, 2 Giralda Farms, Madison, NJ 07940 USA; 5 VetUnion Veterinary Laboratory, Nagatinskaya naberezhnaya, bild 33, 117105 Moscow, Russia

**Keywords:** Dogs, Hard ticks, Ixodidae, Russia, Tick-borne pathogens

## Abstract

**Background:**

Species of Canidae in Russia can be infested with up to 24 different tick species; however, the frequency of different tick species infesting domestic dogs across Russia is not known. In addition, tick-borne disease risks for domestic dogs in Russia are not well quantified. The goal of this study was to conduct a nationwide survey of ticks collected from infested dogs admitted to veterinary clinics in Russian cities and to identify pathogens found in these ticks.

**Methods:**

Ticks feeding on dogs admitted to 32 veterinary clinics in 27 major cities across Russia were preserved in ethanol and submitted to a central facility for examination. After identification, each tick was evaluated for infection with known tick-borne pathogens using PCR.

**Results:**

There were 990 individual ticks collected from 636 dogs. All collected ticks belonged to the Ixodidae (hard ticks) and represented 11 species of four genera, *Dermacentor*, *Ixodes*, *Rhipicephalus* and *Haemaphysalis*. Four most common tick species were *D. reticulatus*, followed by *I. persulcatus*, *I. ricinus* and *R. sanguineus*. *Ixodes persulcatus* ticks were found to be infected with 10 different pathogens, and ticks of this species were more frequently infected than either *D. reticulatus* or *I. ricinus*. *Ixodes persulcatus* females were also more frequently co-infected with two or more pathogens than any other tick. Pathogenic species of five genera were detected in ticks: *Anaplasma centrale*, *A*. *phagocytophilum* and *A*. *marginale*; *Babesia canis*, *B. microti*, *B. venatorum*, *B. divergens*, *B. crassa* and *B. vogeli*; *Borrelia miyamotoi*, *B. afzelii* and *B*. *garinii*; *Ehrlichia muris*, *E. canis* and *E*. *ruminantum*; and *Theileria cervi*. *Anaplasma marginale*, *E. canis*, *B. crassa*, *B. vogeli* and *T. cervi* were detected in *I. persulcatus*, and *Babesia canis* in *D. marginatum*, for the first time in Russia.

**Conclusions:**

Multiple ticks from four genera and 11 species of the family Ixodidae were collected from domestic dogs across Russia. These ticks commonly carry pathogens and act as disease vectors. *Ixodes persulcatus* ticks present the greatest risk for transmission of multiple arthropod-borne pathogens.

## Introduction

Hard ticks are dangerous ectoparasites of domestic dogs because of their blood-feeding, toxicoses (including paralysis), irritation, allergy, and potential pathogen transmission. Many ticks are found in Russia, and over a hundred different tick species of the genera *Haemaphysalis* Koch, *Dermacentor* Koch, *Rhipicephalus* Koch and *Ixodes* Latreille are reported from moderate and subtropical climate zones; of these, 24 species are known to infest Canidae [1, 2]. However, there are no data regarding the species composition of ticks infesting urban dogs in Russia and additional information on tick attachment risks for dogs in Russia is important because of the potential for tick-borne disease spread [3]. In addition, tick populations are growing and their geographical range is changing in association with climate change, leading to tick infestation of naïve populations [4–8]. Tick populations can spread quickly over large distances, facilitated by the mobility of people and their companion animals [9, 10]. Dog owners also face tick-borne disease risks while out walking with their animals. There were 6439 cases of human tick-borne Lyme disease reported in Russia in 2014, or 4.41 cases per 100,000 people [11].

Many organisations in multiple Russian cities need to be coordinated to manage a programme that collects and identifies ticks, and then completes the detection of pathogen presence. However, it is important to record the current geographical distribution of tick- and tick-borne disease risks for dogs and also gain insight into regional zoonotic infection risks. Therefore, the goal of this study was to conduct a nationwide survey of ticks infesting dogs admitted to veterinary clinics in multiple Russian cities. Collected ticks were identified to species and then evaluated for vector-borne canine, and potentially zoonotic, pathogens.

## Methods

### Sample collection and parasitological analysis

Veterinary practices in Russian cities were invited to register for the study through voluntary completion of an on-line questionnaire at a custom web site, now closed. The questionnaire collected data on: location (city); clinic name; sample ID; tick removal date; dog breed; age; clinical signs; and diagnosis. All registered practices were provided with tubes containing 70% ethanol for tick collection and submission.

Veterinarians in participating practices collected attached ticks from dogs presented voluntarily to the clinic by their owners. The veterinarian completed a thorough external physical exam to remove ticks attached to the dog and then selected two ticks for preservation and submission. Each saved tick was placed in a separate tube with 70% ethanol and assigned a unique identification number.

Questionnaire responses were entered into a spreadsheet (Excel, Microsoft, Redmond, WA, USA) published online and the initial 1000 submitted ticks were submitted for pathogen analysis in addition to tick identification. The ticks were identified to the species level using morphological keys and the life-cycle stage and sex were determined [1, 2, 12]. Where tick identification based on morphology was uncertain, sequencing was used; if the species could still not be reliably identified, the tick was excluded from the study. All ticks were then processed for pathogen identification using qPCR.

### DNA extraction, amplification and sequencing

Frozen ticks were homogenized (MagNALyser Instrument, MagNaLyserGreenBeads, Roche Diagnostics, Switzerland) and total DNA was extracted (RealBest Extraction 100, Vector-Best, Novosibirsk, Russia) according to the manufacturer’s protocol.

Oligonucleotide sequences were designed (PrimerQuest online software, Integrated DNA Technologies, USA) and produced (Vector-Best, Novosibirsk, Russia). Real-time PCR was performed (CFX96 thermal cycler, Bio-Rad Laboratories, USA) and tests were manually set-up. Each reaction required 50 μl total volume and included 1× PCR buffer (Vector-Best, Novosibirsk, Russia), 0.4 mM of dNTP (Biosan, Russia), 1% BSA and 1U Taq polymerase (Vector-Best, Novosibirsk, Russia) pre-mixed with active center-specific monoclonal antibody (Takara Bio, Mountain View, CA, USA), 0.5 units of uracil-DNA glycosylase (Vector-Best, Novosibirsk, Russia), 0.5 μM of each primer and 0.25 μM of dual-labeled probe. PCR cycling conditions were: 2 min incubation at 50 °C; 2 min pre-denaturation step at 94 °C; 50 denaturation cycles (94 °C for 10 s) alternated with annealing and elongation (60 °С for 20 s).

For screening analysis, *B*. *miyamotoi* and *B*. *burgdorfgeri* (*s.l*.) DNA was detected by *TaqMan* real-time PCR (RealBest DNA *Borrelia miyamotoi* and RealBest DNA *Borrelia burgdorfgeri* (*s.l*.), Vector-Best, Novosibirsk, Russia) according to the manufacturer’s protocol. *Babesia* spp and *Anaplasma* spp.*/Ehrlichia* spp. were detected by the real-time PCR method, using oligonucleotides for the detection of *Babesia* and *Anaplasma/Ehrlichia*. *Borrelia* spp. were then determined by species-specific qPCR and *Ehrlichia* spp., *Anaplasma* spp., and *Babesia* spp. were identified using sequencing. *Hepatozoon* spp. were not screened for in this study.

PCR products were purified (GFX Columns, Amersham Biosciences, USA) and sequenced (ABI 3500 Genetic Analyzer, Applied Biosystems, USA), aligned (BioEdit v7.2.5, Ibis Biosciences, USA) and analyzed (BLASTN software; http://www.ncbi.nlm.nih.gov/BLAST).

Nucleotide sequences of pathogens detected in Russia for the first time were deposited in the GenBank database under the accession numbers MG028598 (*A. marginale*), MG066535 (*E. canis*), MG062780 (*B. microti*), MG062781 (*B*. *venatorum*), MG062782 (*B*. *crassa*), MG041384 (*B*. *vogeli*) and MG041373 (*Theileria cervi*).

### Statistics

Pathogen prevalence differences in ticks between sites and among pathogens within tick species were computed by the Pearson’s Chi-square goodness-of-fit test, when conditions were met, or otherwise by Fisher’s exact test (IBM SPSS Statistics, version 22; Statistica software, version 12). *P* < 0.05 was considered significant; 95% confidence intervals for tick pathogen prevalence were computed using a bootstrap technique.

## Results

### Tick distribution

**Fig. 1:**
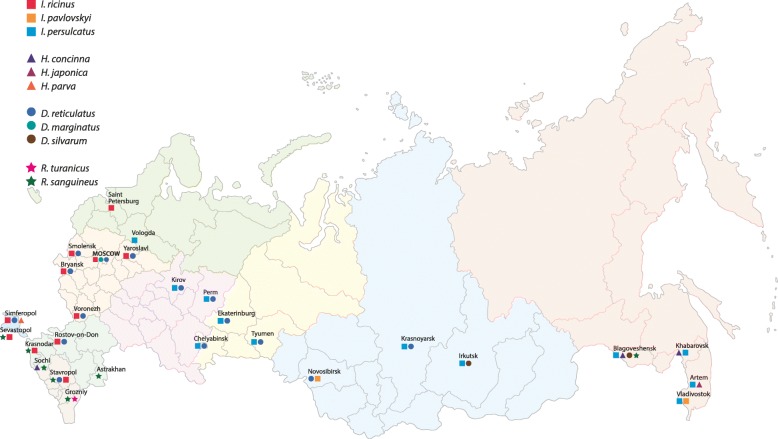
Map of Russia showing the geographical distribution of hard ticks collected from dogs in major cities, 2016

The study was completed in 2016, with participation of 32 veterinary clinics from 27 cities located across Russia (Fig. 1). There were 1010 ticks removed and submitted from 636 dogs. Of these, 990 ticks were identified to species including 11 species of the Ixodidae (Table 2), although 20 submitted ticks could not be analysed because of damage during collection or transport. Submitted ticks were semi-engorged or fully-engorged and most were adults, with 64.9% adult females and 28.7% adult males. Juvenile ticks were 3.9% nymphs and 2.4% larvae.

The most common tick identified on dogs was *Dermacentor reticulatus* with a prevalence of 40.7% (411/990; 95% CI: 37–43%) (Table 1). This tick species was identified in 17 cities, with the westernmost location in Smolensk (54°46'58"N, 32°2'42"E) and the easternmost in Krasnoyarsk (56°0'38"N, 92°51'9"E). *Ixodes persulcatus* ticks were less commonly identified with a prevalence of 23.8% (218/990; 95% CI: 20–30%) (*χ*^2^ = 64.442, *df* = 1, *P* < 0.001). This species was identified in 12 widely separated cities with the westernmost location at Vologda (59°13'14"N, 39°53'30"E). *Ixodes ricinus* ticks (prevalence 13.1%; 130/990; 95% CI: 10–20%) were submitted from 8 cities (Fig. 1) and significantly less often (*χ*^2^ = 37.661, *df* = 1, *P* < 0.01) than *I. persulcatus*. The easternmost locations for *I*. *ricinus* were Moscow (55°45'21"N, 37°37'3"E) and Voronezh (51°39'42"N, 39°12'1"E). The prevalence of *Rhipicephalus sanguineus* was slightly lower than that of *I*. *ricinus* (11.3%; 112/990) (Fig. 1, Table 2) with an easternmost location of Blagoveshchensk (50°17'26"N, 127°31'38"E). Three species of *Haemaphysalis* were identified with a prevalence of 2.5–2.7%. *Haemaphysalis concinna* (27 ticks) was collected in Blagoveshchensk, Khabarovsk (48°28'49"N, 135°4'19"E), and Sochi (43°35'8"N, 39°43'23"E). *Haemaphysalis parva* (25 ticks) was collected in Simferopol (44°56'54"N, 34°6'1"E); and *H*. *japonica* (27 ticks) in Artem (44°56'54"N, 34°6'1"E). Ticks recorded with lower prevalences included *R*. *turanicus* (2.1%; 29 ticks) from Grozniy (43°19'4"N, 45°41'42"E); *Dermacentor silvarum* (1 male and 3 female ticks) from Blagoveshchensk and Irkutsk (52°17'11"N, 104°16'50"E); and, *D*. *marginatus* (3 female ticks) from Moscow. One *I*. *pavlovskyi* female was submitted from Novosibirsk (55°1'49"N, 82°55'14"E), and one from Vladivostok (43°6'55"N, 131°53'7"E).

**Table 1:** Feeding ticks collected from dogs by veterinarians in cities across the Russian Federation

Genus	Species	Number collected (F / M / N /L)^a^	% of total (95% CI)	Location (*n*)
*Ixodes*	*I. ricinus*	130 (122/6/2/-)	13.1 (10–20)	Bryansk (8), Moscow (69), Rostov-on-Don (1), Sevastopol (1), Simferopol (8), Smolensk (3), Stavropol (5), St. Petersburg (14), Voronezh (18), Yaroslavl (3)
	*I. pavlovskyi*	2 (2/-/-/-)	0.2 (0.02–0.7)	Novosibirsk (1), Vladivostok (1)
	*I. persulcatus*	238 (200/21/11/6)	23.8 (20–30)	Artem (13), Blagoveshchensk (3), Chelyabinsk (1), Ekaterinburg (19), Irkutsk (59), Khabarovsk (9), Kirov (51), Krasnoyarsk (30), Perm (6), Tyumen (2), Vladivostok (13), Vologda (32)
*Haemaphysalis*	*H. concinna*	27 (16/9/2/-)	2.7 (2–4)	Blagoveshchensk (10), Khabarovsk (14), Sochi (3)
	*H. japonica*	27 (-/6/3/17)	2.7 (2–4)	Artem (27)
	*Н. parva*	25 (18/7/-/-)	2.5 (2–4)	Simferopol (25)
*Dermacentor*	*D. reticulatus*	404 (231/173/-/-)	40.7 (37–43)	Bryansk (13), Chelyabinsk (12), Ekaterinburg (53), Grozniy (1), Kirov (1), Krasnodar (3), Krasnoyarsk (36), Moscow (57), Novosibirsk (40), Perm (14), Rostov-on-Don (9), Simferopol (45), Smolensk (20), Stavropol (22), Tyumen (65), Voronezh (12), Yaroslavl (1)
	*D. marginatus*	3 (3/-/-/-)	0.3 (0.06-9)	Moscow (3)
	*D. silvarum*	4 (3/1/-/-)	0.4 (0.06–0.9)	Blagoveshchensk (1), Irkutsk (2)
*Rhipicephalus*	*R. turanicus*	29 (11/18/-/-)	2.1 (2–4)	Grozniy (29)
	*R. sanguineus*	112 (42/48/21/1)	11.3 (10–14)	Astrakhan (12), Blagoveshchensk (2), Grozniy (17), Krasnodar (6), Sevastopol (52), Sochi (12), Stavropol (11)
Total		990 (642/284/39/24)		

^a^L, larvae; N, nymphs; F, females; M, males

**Table 2:** Pathogens detected using molecular techniques in ticks collected from dogs in Russia in 2016

Genus	Species	Number (%) PCR positive ticks [95% CI]					
		*I. ricinus* (*n* = 130)	*I. persulcatus* (*n* = 238)	*Н. Parva* (*n* = 25)	*D. marginatus* (*n* = 3)	*D. reticulatus* (*n* = 404)	*Rh. sanguineus* (*n* = 112)
*Anaplasma*	*A. centrale*	0	0	1 (4) [0.1–2.0]	0	0	0
	*A. phagocytophilum*	4 (3.1) [0.9–7.7]	7 (3.0) [1–6]	0	0	1 (0.3) [0.1–2.0]	0
	*A. marginale*	2 (1.5) [0.2–5.5]	0	0	0	0	0
*Ehrlichia*	*E. muris*	0	9 (3.8) [2–7]	0	0	0	0
	*E. canis*	0	0	0	0	0	1 (0.9) [0–5]
	*E. ruminantum*	1 (0.8) [0–4.2]	0	0	0	0	0
*Borrelia*	*B. miyamotoi*	4 (3.1) [0.9–7.7]	2 (0.9) [0.1–3.0]	0	0	2 (0.5) [0.1–2.0]	0
	*B. afzelii*	9 (6.9) [3.2–12.7]	14 (5.9) [3–10]	0	0	0	0
	*B. garinii*	6 (4.6) [1.7–9.8]	35 (14.2) [11–20]	0	0	0	0
*Babesia*	*B. canis*	5 (3.85) [1.3–8.8]	4 (1.69) [1–4]	0	2 (66.7) [9–99]	82 (20.3) [17–25]	0
	*B. microti*	0	2 (0.9) [0–3]	0	0	0	0
	*B. venatorum*	0	1 (0.4) [0–2.4]	0	0	1 (0.25) [0.6–2.0]	0
	*B. divergens*	0	1 (0.4) [0–2]	0	0	0	0
	*B. crassa*	0	0	1 (4) [0.1–20]	0	0	0
	*B. vogeli*	0	0	0	0	0	1 (0.9) [0–4.8]
*Theileria*	*T. cervi*	0	1 (0.4) [0–2.4]	0	0	0	0
Total		31 (23.9) [17–32]	76 (32.2) [26–39]	2 (8) [0.1–2.7]	2 (66.7) [0.9–9.9]	86 (21.3) [17–25]	2 (1.8) [0.2–6.0]

### Pathogen detection

**Fig. 2:**
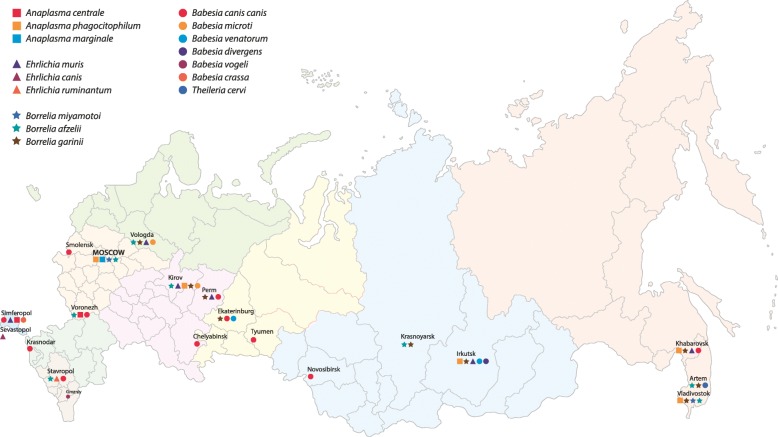
Map of Russia showing the geographical distribution of tick-associated pathogens in major cities, 2016

Sixteen pathogens were detected: 3 *Anaplasma* species; 1 *Ehrlichia* species; *Borrelia miyamotoi*; 2 *Borrelia burgdorferi* (*sensu lato*) genospecies; 6 *Babesia* species; and 1 species of *Theileria cervi*, a cosmopolitan apicomplexan haemoparasite of domestic and wild ungulates (Fig. 2). Pathogen prevalences in all submitted ticks were: *Anaplasma* spp. 1.5% (15/990; 95% CI: 0.8–2.5%); *Ehrlichia* spp. 1.1% (11/990; 95% CI: 0.6–2.0); *Borrelia* spp. 7.3% (90/990; 95% CI: 5.7–9.0); and *Babesia* spp. 10.1% (100/990; 95% CI: 8.3–12.1%). There were 199 ticks (prevalence 20.1%; 95% CI: 17.6–22.7%) from 6 different species in which a single pathogen species was identified (Table 2).

*Anaplasma* spp. prevalence in tick species varied between 0.1–3.1%: *A*. *centrale* was detected in *H*. *parva* (1/25) from Simferopol; *A*. *phagocytophilum* was detected in 3.0% (7/238) of *I*. *persulcatus* and 3.1% (4/130) of *I*. *ricinus*, a non-significant difference (Fisher’s exact test: *P* > 0.05); *A*. *phagocytophilum* was identified in 1 *D*. *reticulatus* (1/404) from Moscow and *A*. *marginale* was detected in 2 *I*. *ricinus* from Moscow.

*Ehrlichia* spp. were detected in 11 ticks with a prevalence between 0.77–3.8% in different tick species. *Ehrlichia muris* was detected in *I*. *persulcatus* only, collected from dogs from widely separated cities including: 3 in Irkutsk; 2 each in Kirov and Vologda; and 1 each in Khabarovsk and Perm. *Ehrlichia canis* and *E*. *ruminantum* were detected in 1 *R*. *sanguineus* from Sevastopol and in 1 *I*. *ricinus* from Stavropol*. Borrelia miyamotoi* prevalence was 0.5–3.1% in tick species submitted. Infected ticks included: 3 *I*. *ricinus* from Moscow and 1 from St Petersburg; 2 *I*. *persulcatus* from Vladivostok; 1 *D*. *reticulatus* from Novosibirsk; and 1 from Simferopol. *Borrelia afzelii* and *B*. *garinii* were only detected in *I*. *ricinus* and *I*. *persulcatus*, with a prevalence between 4.6–14.2%. The overall prevalence of *B*. *burgdorferi* (*s.l*.) in *I*. *persulcatus* (20.8%; 49/238; 95% CI: 15.8–26.5%) was significantly greater (*χ*^2^ = 4.943, *df* = 1, *P* < 0.005) than in *I*. *ricinus* (11.5 %; 15/130; 95% CI: 6.6–18.3%). *Borrelia garinii* (prevalence 11.2%; 41/368; 95% CI: 8.2–14.9%) was detected in *Ixodes* spp. significantly more frequently (*χ*^2^ = 4.658, *df* = 1, *P* < 0.005) than *B*. *afzelii* (prevalence 6.3%; 23/368; 95% CI: 4.0–9.3%). *Borrelia burgdorferi* (*s.l*.) infected ticks were widespread and identified in multiple cities. Cities with few submitted *I*. *ricinus* and *I*. *persulcatus* included: Blagoveshchensk, Bryansk, Rostov-on-Don, Sevastopol, Simferopol and Yaroslavl. There were 6 tick species infected with *Babesia* spp. and prevalence varied from 0.4 to 20.3%. The highest *B*. *canis* prevalence was among *D*. *reticulatus* (Table 3), which were collected from 10 cities. The highest prevalences were in Ekaterinburg (35.8%; 19/53; 95% CI: 23.1–50.2%) and Tyumen (30.8%; 20/65; 95% CI: 20.0–43.4%). Three *D*. *reticulatus* from Krasnodar tested positive for *B*. *canis* and 1 *D*. *reticulatus* from Ekaterinburg was positive for *B*. *venatorum. Babesia microti* was detected in 1 *I*. *persulcatus* from Kirov and 1 from Vologda while *B*. *divergens* was detected in *I*. *persulcatus* from Irkutsk. *Babesia crassa* and *B*. *vogeli* were detected in ticks from Simferopol and Grozniy, respectively. *Theileria cervi* was detected in 1 *I*. *persulcatus* tick from Artem.

**Table 3:** Pathogen co-infections in ticks collected from dogs in multiple cities in Russia

Species	Location	No. tested (F/M/N/L)^a^	Type of co-infection No. (%) ticks infected	95% CI
*Ixodes persulcatus*	Artem	13 (6/-/1/ 6)	*B.g.* + *B.a.*: 1 (7.7)	0.2–36.0
	Ekaterinburg	19 (17/-/2/-)	*B.g*. + *B.a*.: 1 (5.3)	0.1–26.0
	Irkutsk	59 (53/6/-/-)	*B.g*. + *B.v*. + *A.ph*.; *B.g*. + *E.m*.; *B.d*. + *E.m*.: 1 (1.7)	0.9–9.1
			*B.g.*+ *B.a.*: 2 (3.4)	0.4–11.7
	Khabarovsk	9 (9/-/-/-)	*B.g*.+ *A.ph.*: 1 (11.1)	0.3–48.2
	Kirov	50 (38/9/3/-)	*B.a*. + *E.m*.; *B.a*. + *B.mic*.; *B.a*. + *A.ph*.: 1 (2.0)	0.1–10.6
			*B.g*. + *B.a*.: 3 (6.0)	1.3–16.5
	Krasnoyarsk	30 (28/-/2/-)	*B.g.* + *B.a.*: 1 (3.3)	0.1–17.2
	Perm	6 (6/-/-/-)	*B.g.* + *B.a.*; *B.g*. + *E.m*.: 1 (16.7)	0–64.1
	Vladivostok	14 (11/3/-/-)	*B.a*. + *A.ph*.; *B.g*. + *B.m*.: 1 (7.1)	0.2–33.9
	Vologda	32 (27/2/3/-)	*B.g.*+ *B.a.*: 3 (9.4)	2.0–2.5
			*B.a*. + *E.m*.; *B.g*. + *B.a.* + *E.m*.; *B.g*. + *B.a*. + *B.mic*.: 1 (3.1)	0.1–16.0
*Ixodes ricinus*	Moscow	70 (67/2/1/-)	*B.m*. + *A.m*.; *B.a*. + *B.c*. + *A.m*.: 1 (1.4)	0–7.7
	St Petersburg	14 (14/-/-/-)	*B.g*.+ *B.m.*: 1 (7.1)	0.2–33.9
Total		316 (276/22/12/6)		

^a^F, females; M, males; N, nymphs, L, larvae

*Abbreviations*: *A.ph*., *Anaplasma phagocytophilum*; *A.m*., *A. marginale*; *E.m*., *Ehrlichia muris*; *B.m*., *Borrelia miyamotoi*; *B.a*., *B. afzelii*; *B.g*., *B. garinii*; *B.mic*., *Babesia microti*; *B.c*., *B. canis*; *B.v*., *B. venatorum*; *B.d*., *B. divergens*

*Ixodes persulcatus* was the tick species that was most commonly pathogen infected with an infection prevalence significantly greater than *D*. *reticulatus* (*χ*^2^ = 9.282, *df* = 1, *P* < 0.01) although not significantly greater than *I*. *ricinus* (*χ*^2^ = 2.830, *df* = 1, *P* > 0.05). *Ixodes persulcatus* also carried the greatest diversity of detected arthropod-borne pathogens, with 10 different species identified (Table 2).

### Pathogen co-infection in ticks

The prevalence of co-infection in ticks (2.7%; 19/316; 95% CI: 1.8–3.9%), was significantly lower (*χ*^2^ = 167.012, *df* = 1, *P* < 0.01) than the prevalence of a single pathogen infection and only 2 species had co-infections (Table 3). *Ixodes persulcatus* adult females were the most commonly co-infected ticks (Fisher’s exact test: *P* < 0.05) and 21 (prevalence 8.9%; 95% CI: 5.6–13.3%) were infected with more than one pathogen including *A*. *phagocytophilum*, *E*. *muris*, *B*. *miyamotoi*, 2 genospecies of *B*. *burgdorferi* (*s.l*.), *B*. *microti* and *B*. *divergens* (Table 3). Three female ticks had a triple infection involving *A*. *phagocytophilum*, *B*. *burgdorferi* (*s.l*.), *B*. *microti*, and *B*.*venatorum* (prevalence 1.3%; 95% CI: 0.3–3.7%). Co-infections with *B*. *burgdorferi* (*s.l*.) and another pathogen were common among *I*. *persulcatus* ticks, while double infections with *E*. *muris* and *B*. *divergens* were observed only once. *Ixodes ricinus* females co-infected with 2 or more tick-borne pathogens were collected from 2 cities (Table 3) and pathogens involved were *A*. *marginale*, *B*. *miyamotoi*, *B*. *afzelii*, *B*. *garinii* or *B*. *canis* in various combinations.

## Discussion

This is the first comprehensive study of ticks found attached to domestic dogs and their associated tick-borne pathogens conducted in multiple Russian cities. The results show that there are a wide variety of infesting ixodid ticks and that there are multiple potential pathogens that can be transmitted through a tick bite, with a frequent risk of more than one pathogen from each tick. This result is generally consistent with observations on ticks and pathogens from dogs in other countries, although there are some unique results in this study.

The most common tick species from domestic dogs in Russian cities were *D*. *reticulatus*, *I*. *persulcatus* and *I*. *ricinus*. In this study, *D*. *reticulatus*-infested dogs were found in the widest geographical range, with reports from Smolensk to Krasnoyarsk. Previously, the distribution of *D*. *reticulatus* was linked to the southern taiga in the eastern part of the geographical range and the eastern limit of distribution was previously reported as the upper reaches of the River Yenisei [2]. *Ixodes persulcatus* had the second greatest prevalence in urban dogs in this study although ticks were not found on dogs outside the tick’s previously described geographical range. However, there have been reports of *I*. *persulcatus* to the south and east of the main geographical range [13, 14]. *Ixodes ricinus* ticks were submitted from dogs in 8 cities. This tick is known to be widely distributed in Russia; it inhabits European deciduous and mixed forests and can be found in the subregions of southern and sometimes northern taiga [1]. Additional tick genera collected from dogs in this study included species of *Haemaphysalis*, *Dermacentor* and *Rhipicephalus*. *Rhipicephalus* spp. ticks were submitted in a similar proportion to *I*. *ricinus*; however, unlike the more widely distributed *I*. *ricinus*, *Rhipicephalus* spp. were primarily found on dogs from cities in humid and arid subtropical regions, although *R. sanguineus* was recovered from the northeastern city of Blagoveshchensk (Fig. 1). *Rhipicephalus sanguineus* is a globally widespread tick that preferentially infests dog hosts and is also called the “kennel” or “brown dog” tick [15]. *Rhipicephalus sanguineus* in southern Italy has induced tick paralysis outbreaks in dogs [16]. *Rhipicephalus turanicus* was submitted from Grozniy in this study, and although this tick was previously reported in Russia and the former USSR, available information is fragmentary and collected in the mid-20th century [2]. This tick is morphologically similar to *R*. *sanguineus* [17] but has very different behavioural, ecological and vector characteristics [18]. The present study contributes new information about the risks of *H*. *concinna* and *H*. *japonica* infestation of dogs in eastern Russia. *Haemaphysalis concinna* is found in the moderate climate zone of Eurasia and is common in China and Mongolia [19, 20]. *Haemaphysalis japonica* infests dogs in Japan, Mongolia and China [21–23] and *H*. *parva* is also known as a parasite of dogs [24]; however, this is the first known report of *H*. *parva* on dogs in Russia. The previous limited experience with these two tick species on dogs in Russia could reflect a bias toward examination of domestic dogs in urban biotopes, where there is a lower risk of encountering these species of ticks. However, growth of cities, increased tourist movement, and a greater opportunity for owners to walk dogs in rural areas, may lead to more frequent encounters with these tick species. *Haemaphysalis parva* feeds on a wide range of large mammals and it is likely that this tick was not reported previously on dogs in Russia because there are few previous investigations on the spectrum of ticks parasitizing Russian dogs. The possibility that this tick plays a significant role in vector borne disease transmission to dogs where it is endemic requires further investigation. Three female *D*. *marginatus* were collected from dogs in Moscow, and this thermophilic species is reported to be widespread in the Mediterranean region. In Russia, *D*. *marginatus* is widely distributed in the south, while the geographical range in the east reaches the southern forest steppes of Western Siberia. *Dermacentor silvarum*, similar to *D*. *marginatus*, prefers forest steppes and is common in eastern Russia, Mongolia and China [23–25]. These tick species are uncommon in ecosystems transformed by human activity, which likely explains their low prevalence in this study of dogs from urban areas.

Ticks in this study were feeding on dogs at the time of collection and were therefore potentially transmitting any carried pathogen to the dogs while feeding. Detected pathogens are also an indication of the tick-transmitted pathogens circulating in the local community. Specific tick species represent a greater risk for some pathogens, for example, pathogens were detected in *D*. *reticulatus* at variable prevalences: *B*. *canis* (20.3%); *B*. *miyamotoi* (0.5%); *A*. *phagocytophilum* (0.25%); and *B*. *venatorum* (0.25%). Previous studies show that the most significant and widespread pathogen transmitted by *D*. *reticulatus* ticks is *B*. *canis* which is found from western Europe to Siberia [26, 27] and the present study confirms that these ticks and pathogens are widespread across Russia. Veterinarians practising in Khabarovsk in eastern Russia (Fig. 1) have diagnosed canine babesiosis although no ticks from this city were *B*. *canis-*positive indicating that the vector and specific pathogen in this region are not yet identified. Field-collected *D*. *reticulatus* ticks from prior years and from other areas of Europe have had *B*. *canis* infection prevalence between 0.7–15.0% [28]. In contrast, little is known about the significance of *D*. *reticulatus*-transmitted *B*. *venatorum* in Russia. *Ixodes persulcatus* and *I*. *ricinus* are believed to be the main vectors for the transmission of *B*. *venatorum* [29]. *Babesia gibsoni* DNA was not detected in any ticks in this study; however, this pathogen has been reported in dogs from Moscow, the Lipetsk Oblast, Ufa, St Petersburg and Oryol [30].

*Ixodes persulcatus* ticks carried the widest array of tick-borne pathogens, including *A*. *phagocytophilum* and *E*. *muris* detected in ticks submitted from seven cities. The zoonotic cycles of these pathogens are established in these locations and these *Anaplasmataceae* pathogens were previously detected using molecular methods in unfed *I*. *persulcatus* in several regions in Russia [31]. The prevalence of *I*. *persulcatus* with *Borrelia* spp. DNA in this study was 21.6%, a value similar to a previous report of 14.9% prevalence of *Borrelia* spp. in *I*. *persulcatus* [32]. *Ixodes persulcatus* are known to be primary carriers and reservoirs for *B*. *burgdorferi* (*s.l*.) and *B*. *miyamotoi* in Russia [33]. Where it is present, *I*. *persulcatus* is known as the main source for these pathogens for both humans and companion animals. *Babesia canis* DNA was detected in one *I*. *persulcatus* from Ekaterinburg and this adult female may have ingested the pathogen while feeding on the dog, which was reported to be showing clinical signs of babesiosis with a positive blood smear for *B*. *canis*. *Babesia microti*, *B*. *venatorum* and *B*. *divergens* DNA were detected in *I*. *persulcatus* collected from dogs in Irkutsk and Kirovin this study, while *B*. *microti* and *B*. *venatorum* DNA were reported in a previous study in *I*. *persulcatus* ticks collected from vegetation in the Russian Far East [31]. *Ixodes persulcatus* was proposed as the main vector for babesiosis in China [34]. *Theileria cervi* DNA was detected in one *I*. *persulcatus* in the eastern city of Artem and *T*. *cervi* was previously reported from white-tailed deer blood and *I*. *scapularis* in North America [35].

*Ixodes ricinus* were second to *I*. *persulcatus* in the prevalence and diversity of pathogens carried, with *A*. *phagocytophilum* and *A*. *marginale* DNA detected. *Ixodes ricinus* is known as the major vector for *Anaplasma* spp. in Europe [36]. *Ehrlichia ruminantium*, the pathogen that causes “heart water” disease in cattle, sheep and goats [37], was detected in one *I*. *ricinus* from Stavropol. The mean prevalence of *I*. *ricinus* infected with one *Borrelia* pathogen, including *B*. *miyamotoi* or two *Borrelia* genospecies was 11.5%, and this tick is reported to be the main vector of *B*. *miyamotoi*, *B*. *afzelii* and *B*. *garinii* in western Russia [38]. The prevalence in the present study is similar to the 13.9% mean prevalence of *B*. *burgdorferi* (*s.l*.) reported in female *I*. *ricinus* in Europe [39]. Five *I*. *ricinus* ticks in this study were positive for pathogens that cause canine babesiosis, although these ticks are not considered to be a vector for canine babesiosis [40]. It is possible that these ticks ingested Babesia infected canine peripheral blood cells from infected dogs leading to subsequent pathogen DNA detection. *Rhipicephalus sanguineus* were positive for *E*. *muris* DNA (Sevastopol) or *B*. *vogeli* (Grozniy). *Babesia vogeli* DNA was previously reported in *R*. *sanguineus* in the Rostov district [41] and experiments have shown that *R*. *sanguineus* transmits *Babesia* spp. (e.g. *B*. *vogeli*) to dogs. Confirmation of *B*. *vogeli* in this study convincingly demonstrates the presence of this pathogen in Russia although its distribution in Russia is not well known. *Babesia vogeli* and *E*. *muris* are pathogens that could pose a serious danger to animals and their detection in ticks attached to dogs is also an indicator of a possible infection risk to the dog owner.

This study found previously unreported tick infections in Russia: *B*. *crassa* was detected in *H*. *parva* in Russia for the first time, while previously, *B*. *crassa* DNA was only reported in *H*. *parva* attached to humans in Ankara, Turkey [42]; and *B*. *canis* DNA was also detected for the first time in two *D*. *marginatus* from Moscow.

The true prevalence of co-infections with human pathogens among hard ticks in most regions of Russia is unknown [43, 44]. In this study, the risk of tick co-infection with more than one pathogen was found to be greatest in regions where *I*. *persulcatus* is the predominant tick. This tick was submitted from veterinary practices in nine cities and *I*. *persulcatus* ticks with more than one pathogen were primarily submitted from known borreliosis-endemic areas. *Ixodes persulcatus* co-infected with *B*. *burgdorferi* (*s.l*.) and two species of the *Anaplasmataceae* were submitted from six cities across Russia. However, results of multiple previous studies indicate that in most regions a minority of *Ixodes* ticks are co-infected [45]. The present study found *B*. *venatorum* and *B*. *divergens* (0.42%) infection hotspots in Irkutsk; however, these pathogens were only detected in co-infection with zoonotic bacteria. Two ticks co-infected with *B*. *microti* (0.85 %) and *B*. *garinii* or *B*. *afzelii* were reported, one each in Kirov and Vologda. *Babesia microti* co-infections with *Borrelia* spp. may increase the risk of babesiosis in Lyme disease endemic areas [46]. Ticks with three co-infecting pathogens were submitted from Irkutsk and Vologda, where *Babesia* spp. DNA was detected in combination with *B*. *burgdorferi* (*s.l*.) and two *Anaplasmataceae* spp. Overall, these results reflect the complicated tick-borne pathogen epizootology reported throughout the wide geographical range of *I*. *persulcatus* [22, 47, 48]. These ticks therefore represent a pathogen transmission risk for both dogs and humans [49] and results of this study confirm the risk of tick-borne disease transmission from *I*. *persulcatus* in Russia.

Single *I. ricinus* co-infected with pathogens were detected in two cities, Moscow and St Petersburg, and these co-infected ticks always had *Borrelia* spp. pathogens. Previous studies in Russia reported *I*. *ricinus* with *B*. *burgdorferi* (*s.l*.) genospecies, *A*. *phagocytophylum*, *Rickettsia monacensis* and *R*. *helvetica* [50]. The present study indicates the potential presence of a wider selection of pathogens, including a tick found to have three different pathogen infections therefore underlining the risk for transmission of multiple pathogens to canine hosts with every feeding tick.

In general, owners of dogs need to be made aware of the risks of tick infestation that they face during walks. Pathogens carried by ticks can infect both dogs and people and monitoring of ticks and the pathogens they carry provides insight into the occurrence and spread of zoonotic diseases. Veterinarians in all areas of Russia should keep these risks in mind and educate owners regarding the risks as well as developing optimal approaches for tick protection protocols that maximize owner compliance.

## Conclusions

Multiple ticks from four genera and 11 species of the family Ixodidae were collected from domestic dogs across Russia. These ticks commonly carry pathogens and act as disease vectors. *Ixodes persulcatus* ticks present the greatest risk for transmission of multiple arthropod-borne pathogens. New tick pathogen transmission risks identified in Russia are *B*. *crassa* infecting *H*. *parva* and *B*. *canis* in *D*. *marginatus*.
